# Supplementary figures and images for: Importance of missingness in baseline variables: A case study of the All of Us Research Program
Source: PLoS One. 2023 May 18;18(5):e0285848. doi: 10.1371/journal.pone.0285848 (PMC10194909; doi:10.1371/journal.pone.0285848)

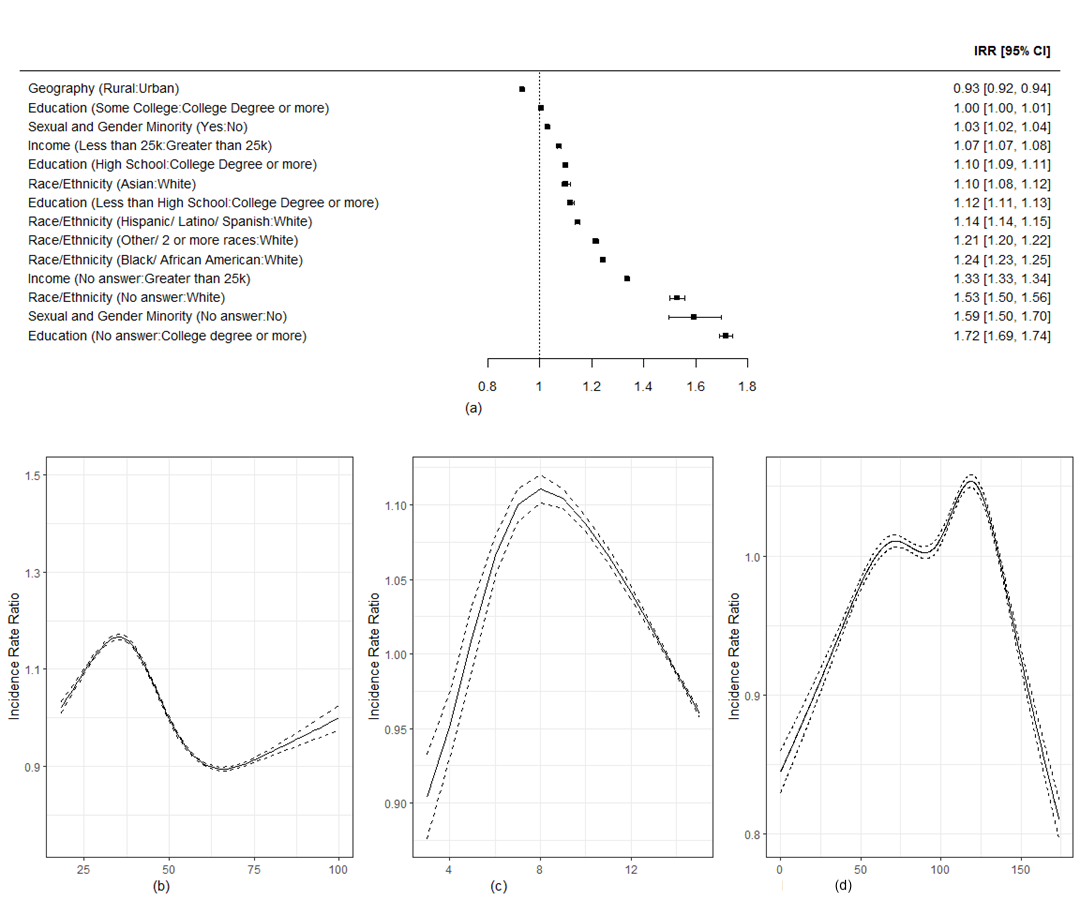

Supplement: S1 Fig — (PNG) [file pone.0285848.s001.png]

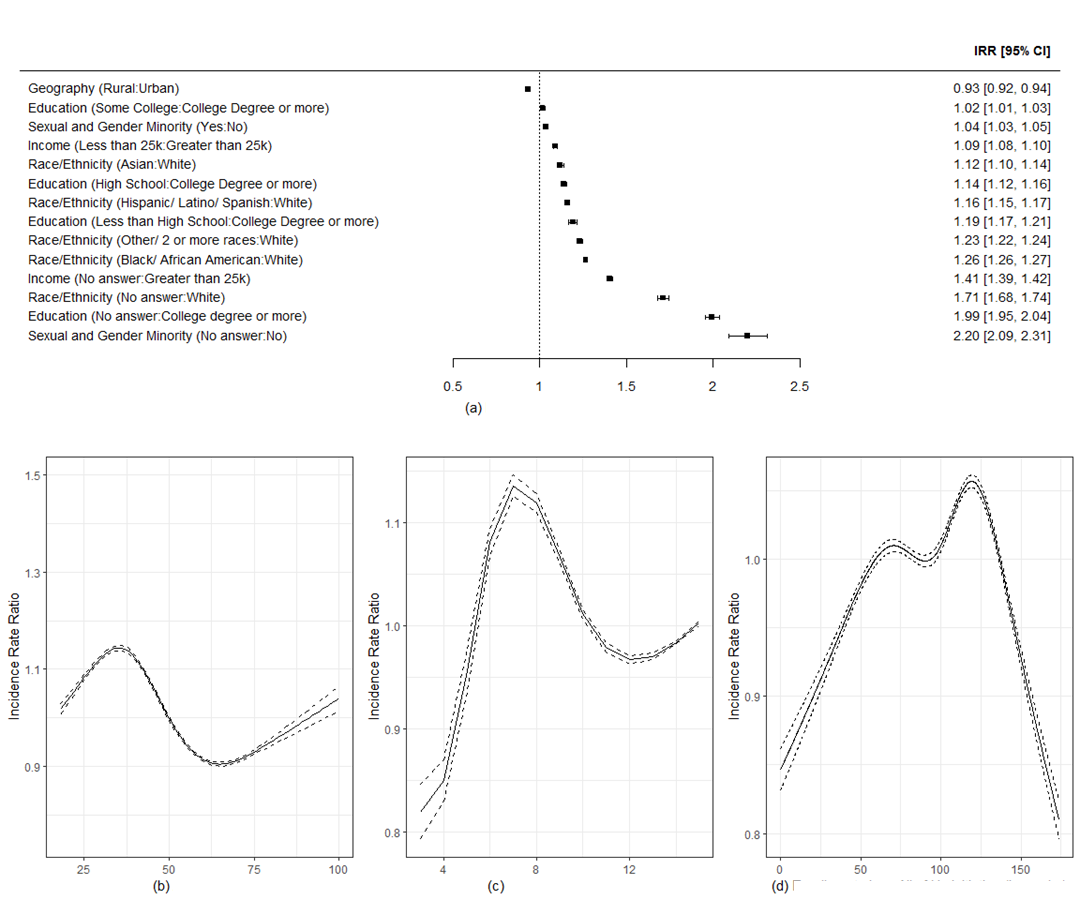

Supplement: S2 Fig — We applied multiple imputation on the total health literacy score. Then we repeated the negative binomial regression on the imputed dataset. (PNG) [file pone.0285848.s002.png]

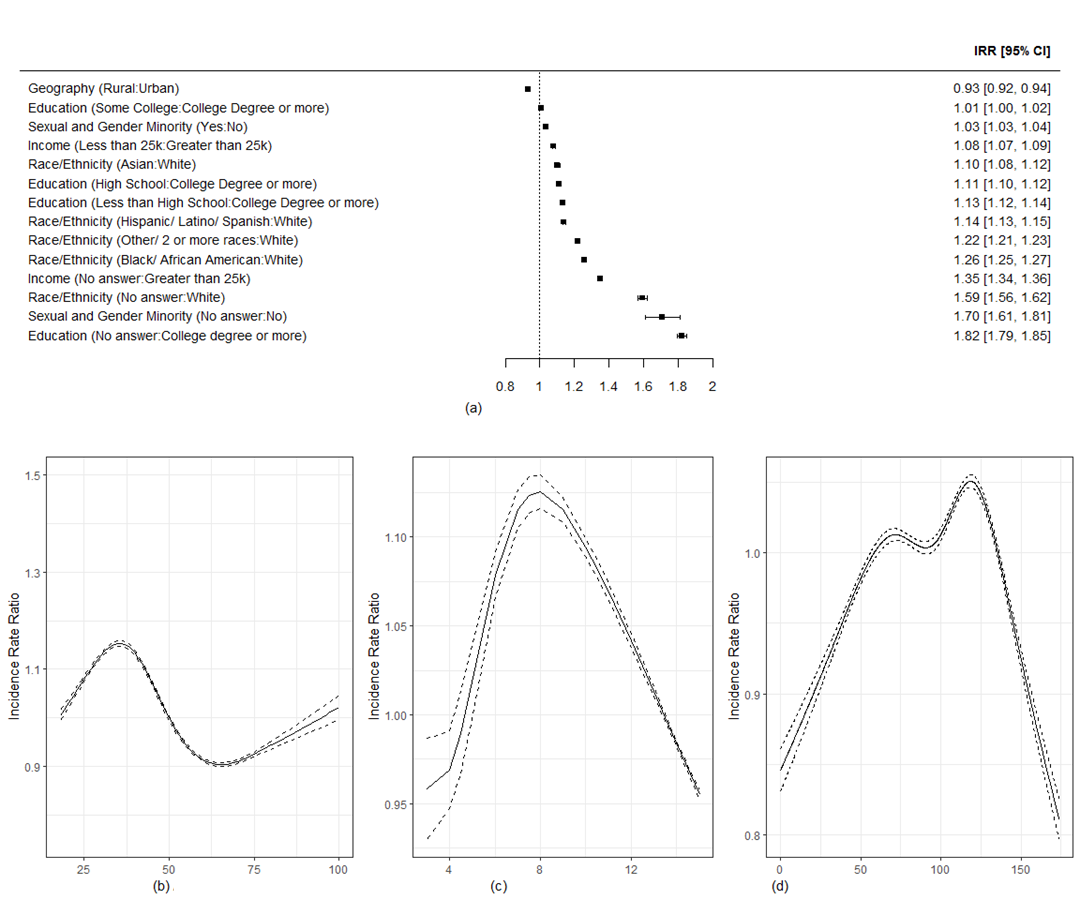

Supplement: S3 Fig — If participants missed only one health literacy score question, we used the average of the two non-missing scores to impute the missing health literacy score. Then we applied negative binomial regression with the complete cases. (PNG) [file pone.0285848.s003.png]

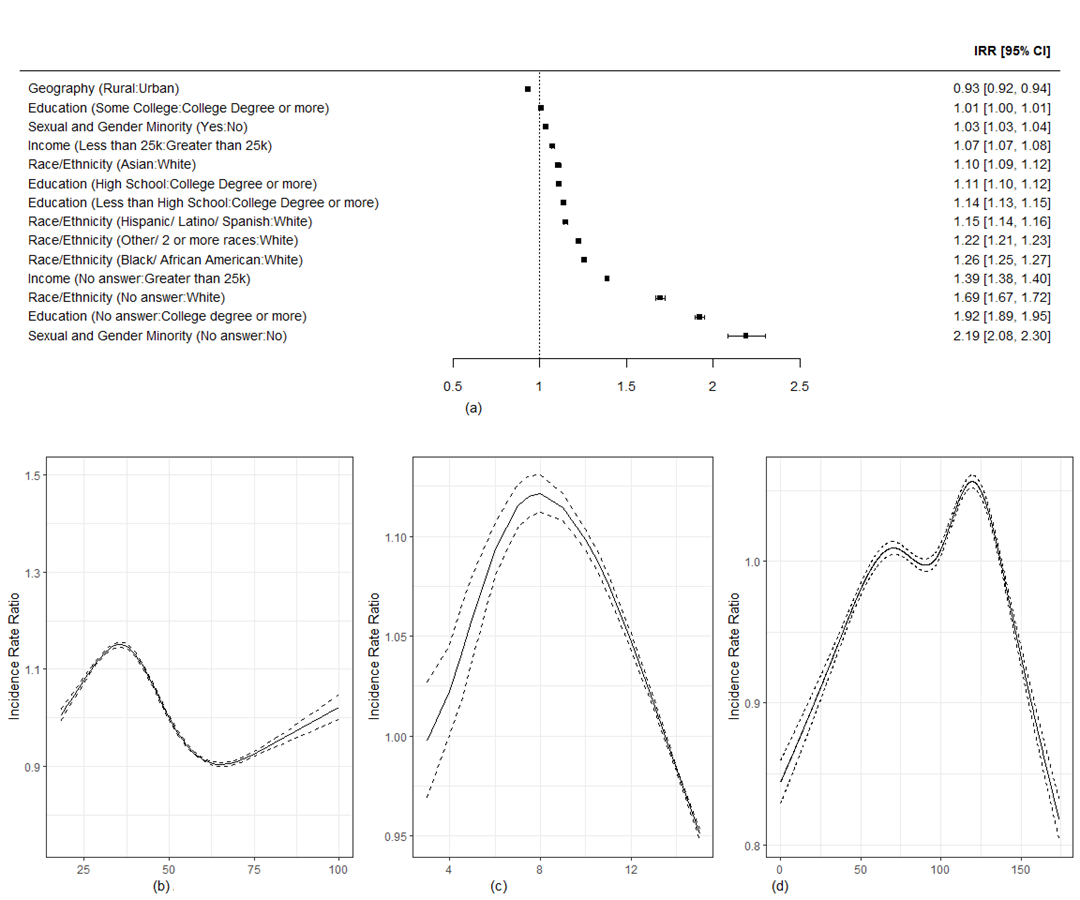

Supplement: S4 Fig — If participants missed only one health literacy score question, we used the average of the two non-missing scores to impute the missing health literacy score. Then we applied multiple imputation on the total health literacy score and repeated negative binomial regression on the imputed dataset. (PNG) [file pone.0285848.s004.png]

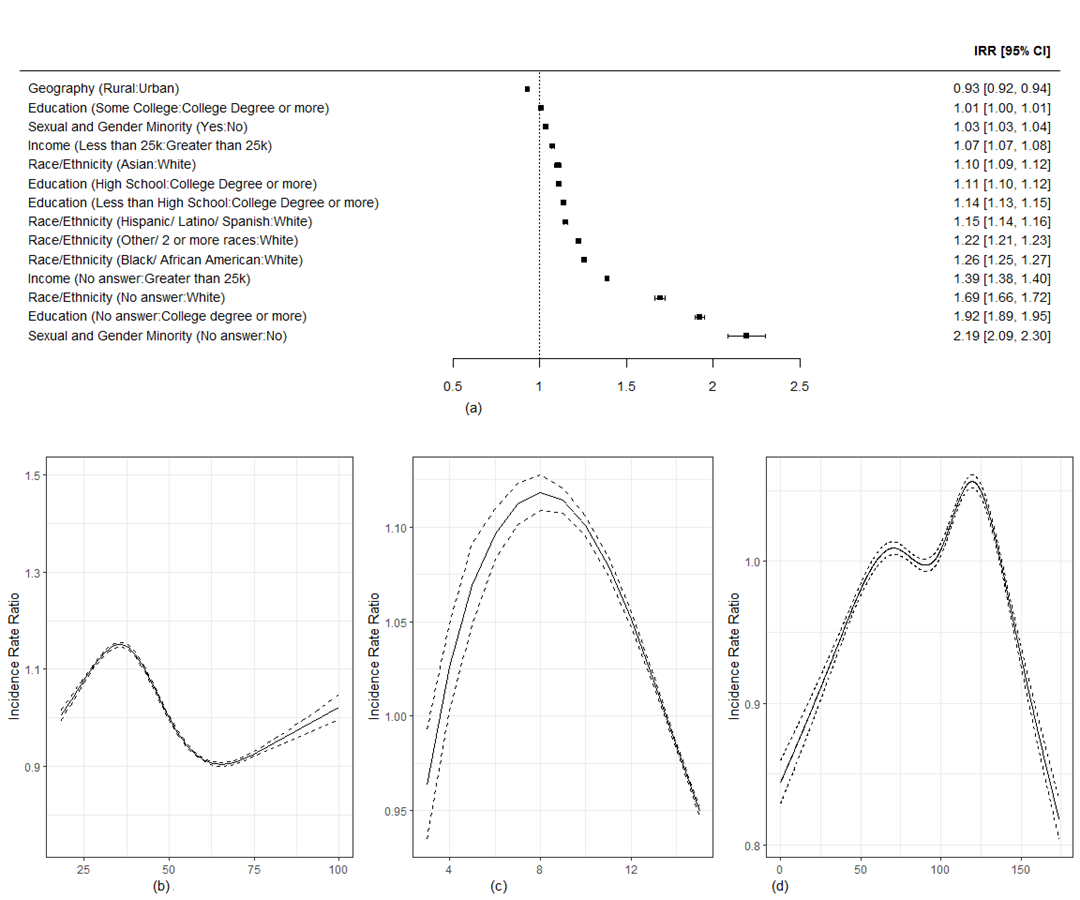

Supplement: S5 Fig — Multiple imputation was applied to health literacy as above, and the negative binomial regression method was applied on the imputed dataset. (PNG) [file pone.0285848.s005.png]

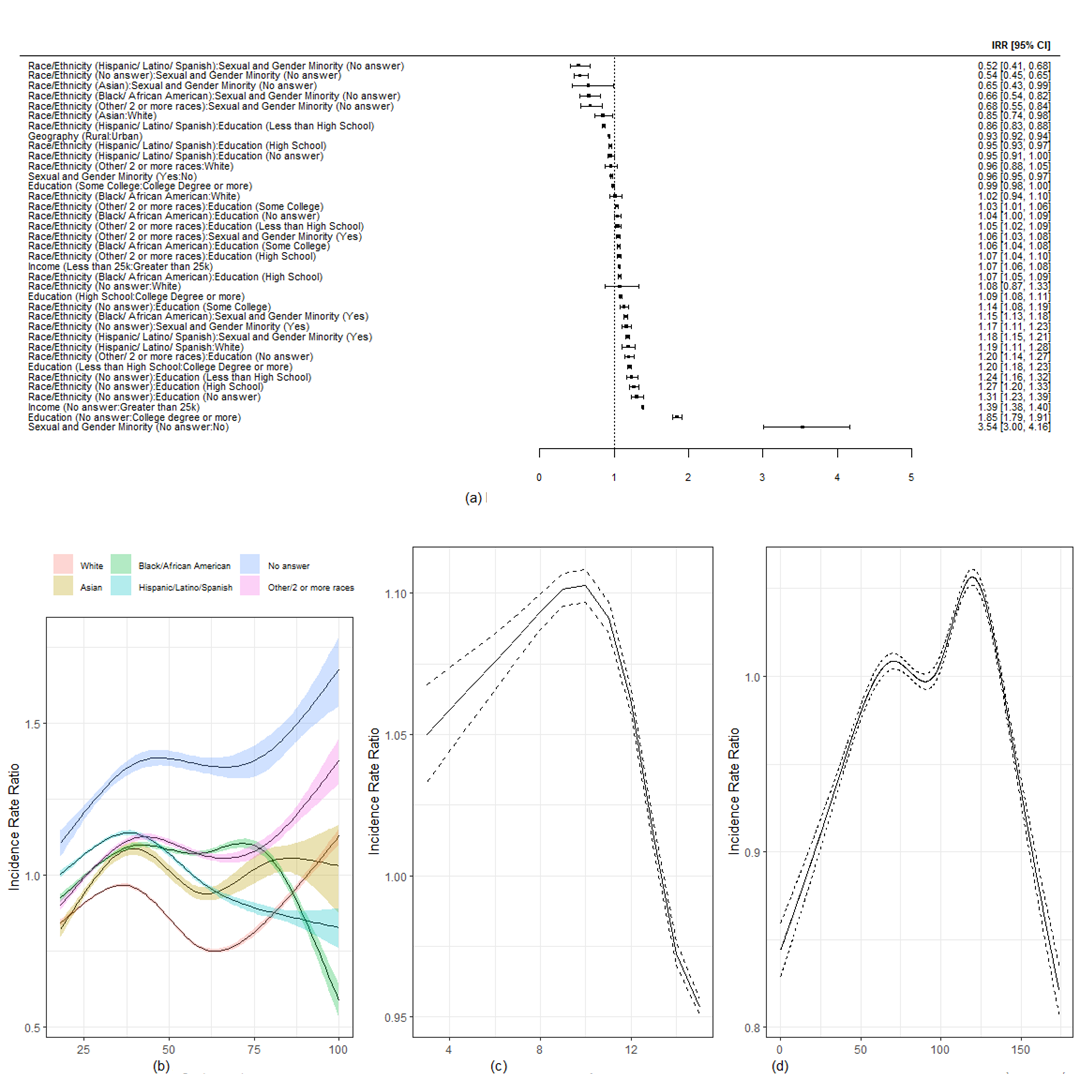

Supplement: S6 Fig — Race/ethnicity interactions with sex/gender, age, and education were added to the model, significant interaction terms were shown in the forest plot. (PNG) [file pone.0285848.s006.png]
