# Supplementary material for: Importance of missingness in baseline variables: A case study of the All of Us Research Program
Source: PLoS One. 2023 May 18;18(5):e0285848. doi: 10.1371/journal.pone.0285848 (PMC10194909; doi:10.1371/journal.pone.0285848)
Supplement: Attachment — Submitted filename: Review_PONE.docx [file pone.0285848.s007.docx]

General comment:

After reading the paper I thought: what is the goal, and what is the core message from the paper? If I understand correctly, the authors are trying to predict whether values on specific questions are missing, using other variables as predictors. Although that may be an interesting thing to look at, I think it would be way too limited to dedicate a complete paper to. Normally, checking whether missing data show a systematic pattern is part of the statistical analysis. I don’t see any statistical analysis here, apart from the prediction of the missing values.

Detailed comments:

1. P. 1, last line: “Various methods…”: This sounds kind of repetitive since earlier it was said that “There are multiple ways to handle missing data in general”. Maybe it should be emphasized more that here you are specifically talking about survey data.
2. P. 4, line 2: “Missing data can be examined by the participant”: at first it read like the examining of the missing data was done by the participant. Please, reformulate.
3. P. 5, line 4: although the advice used to be to impute 5 times, it was later established that 5 imputations is actually quite a small number (see, e.g., Graham, Olchowski, and Gilreath, 2007). I would advice increasing the number of imputations to 100.
4. P. 5, line 7: “Rubin’s rules” needs a reference.
5. I don’t understand the negative binomial regression, nor its purpose. If I understand correctly, a number of predictors is used to predict whether a value on an eligible question is missing or not. These predictors have missing data as well, and are multiply imputed as well? And why is prediction of missing data on the basis of background variables important?
6. I think the layout of the figures is occasionally sloppy. For example, in Figure 1, one of the two categories of age is labeled “>= 65” rather than “≥ 65”, in Figure 2 the variable names overlap with the graph, and “(a) Incidence Rate Ratio” overlaps with the numbers on the X axis.

References:

Graham, J.W., Olchowski, A.E., & Gilreath, T.D (2007). How many imputations are really needed? Some practical clarifications of multiple imputation theory. *Prevention Science, 8,* 206-213. doi: 10.1007/s11121-007-0070-9
