# Supplementary material for: Importance of missingness in baseline variables: A case study of the All of Us Research Program
Source: PLoS One. 2023 May 18;18(5):e0285848. doi: 10.1371/journal.pone.0285848 (PMC10194909; doi:10.1371/journal.pone.0285848)
Supplement: Attachment — Submitted filename: Review_PONE_2.docx [file pone.0285848.s009.docx]

Review of the revision of the paper entitled: “Importance of missingness in baseline variables: a case study of the All of Us”

I think the paper has improved a lot. I do have my doubts however, about the impact this work will. I wonder to what extent far reaching conclusions can be drawn about missing data in such surveys on the basis of just one dataset. However, I’ll leave it up to the editorial board to decide whether the impact is high enough in order for the paper to be publishable. One small textual comment: p. 1, lines 1-2 of second paragraph: “Since health surveys rely on voluntary participation”: is there any research where participation is not voluntary? Please, reformulate.
